# The bewitchment of our intelligence: Scepticism about other minds in anthropology

**DOI:** 10.1177/14634996221080578

**Published:** 2022-02-17

**Authors:** Marco Motta

**Affiliations:** Anthropology Institute, 27214 University of Neuchâtel, Neuchâtel, Switzerland

**Keywords:** Scepticism, doubt, witchcraft, language, violence

## Abstract

This article aims at characterizing how the problem of scepticism about other minds appears in anthropology. To do so, I offer a close reading of Nils Bubandt's book, *The Empty Seashell* (2014), a study of witchcraft and doubt on the North Maluku Island of Halmahera. Through its deep engagement with issues revolving around scepticism, I take the book to be an example of the tendency to consider the problem of sceptical doubt about others as a problem of access to the inner thoughts and feelings of other people. By looking closely at its attempts to respond to this problem, I endeavour to shed light on the ways in which, in working the problem of scepticism out, we may be doing exactly the reverse: giving into the sceptical impulse. How does a certain way of asking questions about scepticism nourish the drive to it? I am interested in the drift towards scepticism that precisely takes the form of a claim against it. In showing that such a drift is prompted by a certain use of language, I hope to elucidate some ways in which scepticism is lived and is thus not merely an intellectual conundrum, but an ordinary human condition.

‘A sceptical anthropologist who does not quite see a witch in the shape of a dog:

this is as close a match to the uncertainties that characterize Buli

witchcraft as I can think of.’

Nils Bubandt, *The Empty Seashell*, 2014, p. xiv

‘Philosophy is a battle against the bewitchment

of our intelligence by means of language.’

Ludwig Wittgenstein, *Philosophical Investigations*, 1953, § 109

## Introduction

For many years now, the problem of scepticism has kept me busy. I began discovering it first during my PhD fieldwork in Zanzibar regarding the kinds of relationships humans have with spirits. Different sorts of doubts pervade these relations in ways that can become damaging for those involved. I progressively came to understand that what anthropology calls ‘witchcraft’ or ‘sorcery’, and Zanzibaris *uchawi*, is, among other things, a form given to corrosive doubts. Later, as I was conducting my postdoctoral research on informal ways of handling social conflicts in Haiti – ways that include what Haitians call *ekspedisyon lanmò*, a sending of zombifying powers, or *koud poud*, a burst of destructive powder – I discovered how some doubts could become deadly. Scepticism kills. Yet this is so because it is *lived*.

Back home, a book waited on my shelves for a long time, but I had never managed to plunge into it until recently: Nils Bubandt's *The Empty Seashell: Witchcraft and Doubt on an Indonesian Island* (2014). The subtitle had caught my attention. As it happens, during the long days of the first confinement in 2020, I opened it and read it through almost at once. I was captured by the details of the descriptions, almost entranced imagining what the author was recounting. I admired the detailed ethnography and the depth of the historical insight. Yet, I was disquieted by something I could not fully grasp; something that has to do with the language, i.e., the way the author formulates the problem emerging from the doubts he is concerned with. I felt this was an opportunity to examine more precisely what it was that troubled me. Hence, I started to write initially without intending to publish any of this, but it turns out that the issues became important enough to compel me to render them public. It finally became this full-length article that is itself a diagnosis of my unease with how the problem of scepticism appears in the book. And since I want to prevent the following considerations from falling flat, I will avoid cutting off what gave birth to them: my discomfort, my musings, my hesitations.

Based on many years of fieldwork and deep knowledge of the historical forces at play in the region, the book explores what it is to live in a world where people are said to turn into cannibal witches, named *gua*. And yet, people are said to be generally doubtful about it. More precisely, *The Empty Seashell* presents itself as an epistemology and ontology of (the uncertainties about) witchcraft in a region where profound social and historical changes have generated much violence. Bubandt is in fact bewildered by what he witnesses: ‘attacks happen – incomprehensibly – within each village: neighbours attack neighbours; kin attack kin; friends attack friends – without any predictability’ (2014: 4). He is even more troubled as he perceives ‘the dangers of the other within oneself’, which in a sense ‘exposes naked identity and makes its maintenance as a safe interior space impossible’ (7). The book thus deals with what it names the ‘contradiction’ between the ‘undeniable reality’ of the *gua* and its ‘impossibility’. Its central concern is the perception that ‘as cannibal witchcraft is radically corporeal, it stubbornly remains existentially opaque’ (3). The book's main target is Evans Pritchard's theory of beliefs, against which it provides an alternative view by claiming that ‘witchcraft is not an object of belief but an experiential aporia’ (xiv).

Bubandt's book brings about a particular inflexion of the problem of scepticism that is worth paying attention to. It is an example of how, in working the problem out, we may be doing exactly the reverse: giving into the sceptical impulse. How does a certain way of asking questions about scepticism nourish the drive to it? Could it be that one yields to scepticism by trying to counter it? I purposely chose a handful of scenes from this book, as well as a small collection of texts directly related to Bubandt's book (Keane, 2016; Morris, 2016; Pelkmans, 2016; Sanders, 2016) instead of an extensive corpus, in order to enable close attention to detail. Because descriptions exceed what has already been said about them, they can also be read in multiple ways. I will thus propose reading the book somewhat against the grain. This is made possible by its remarkable abundance and quality of ethnographic details. Thus, I would like to explore how we read our own ethnographic material. How do our theories derive from, or muscle into, our first-hand material?

The book illustrates not only the intrinsic difficulty of responding to the pressure reality puts on our thinking but also the broader tendency to consider that the problem of sceptical doubt about others is ‘that it is difficult or impossible to know the inner thoughts and feelings of other people’ (2014: 182). It seems to me that this kind of formulation of the problem – that the problem is one of accessibility to the inside of the other – feeds the sceptical doubt it hopes to defeat. This way of formulating the problem shows how captive we are of a certain conception of the relation between the inside and the outside or, if you will, the private and the public.

We, human beings, do not doubt all the time about everything, but we do often doubt, and about all sorts of things. Ordinarily, we may doubt that we have turned off the oven when we leave home, whether tomorrow it will rain, or about the sincerity of a compliment. We may also have doubts about our beliefs. Sometimes, reality itself seems doubtful when we have the feeling that what we experience is not quite real, but a dream perhaps, or just a mirage. A doubt can be small, strong, nagging, funny, lingering, pervasive, haunting, founded or unfounded. In another register, doubt can also be deadly: Othello's doubts about Desdemona's trustworthiness made him kill her, and subsequently himself. All forms of doubt are aspects of our relation to reality that has been turned into a problem coined by philosophers in the West as scepticism.

The history of philosophy is in many ways a history of our interest in human doubt, thus also a history of our disappointment by our responses to scepticism. This is maybe why much of that history is about how particular philosophers reply to sceptical drifts by trying to counter their effects. Concerns of a sceptical nature are found in many traditions of thinking, under many forms. Most famously, the question of whether I am dreaming, thus whether reality is not merely a dream or an illusion (whether there is something out there that exists and is not a mere projection of my mind), has played a crucial role for thinkers as diverse as Zhuangzi, Nāgārjuna, al-Ghāzāli and Descartes^1^. Moreover, the inflection of the problem of scepticism in Western philosophy is twofold: one set of problems emerges when our concern is whether the objects of the external world exist; another set of problems arises when we doubt our knowledge of the other. The first type of scepticism is the matter of concern that we call traditional epistemology, especially since Descartes took up the Pyrrhonean attitude and arguments to overcome them (Machuca and Reed, 2018; Popkin, 2003). The second is commonly known as the ‘problem of other minds’ (Austin, 1946; Avramides, 2019; Cavell, 1999; Gomes, 2018).

One is said to be sceptical about others’ minds when one doubts one's own knowledge of others – of their desires, thoughts, intentions, feelings or beliefs – to the point that one takes the inner life of others to be inaccessible, unattainable. The problem arises when one, like Lily Briscoe in Virginia Woolf's *To the Lighthouse* (Nussbaum, 1995), profoundly desires to get to know others but takes human beings to be sealed off from one another. The tension, then, is between a profound wish to know the other and the perception of the impossibility of that knowledge. In what follows, I aim to characterize how such a problem appears in anthropology.

Scholars from different generations and with considerably different approaches have tackled the problem^2^. Some deal with the difficult task of characterizing the particularities of the scepticism that concerns other human beings (and not just any object of the world), whereas others have the tendency to treat the problem of knowing the other as a general problem of knowledge; that is, often to mistake scepticism about other minds for scepticism about the external world. As I will show below, the confusion between the two is a characteristic feature of the sceptical impulse itself. The consequence, then, is that by imagining that the problem is essentially one of knowing, and because we are used to demarcating territories of inquiry (logics, politics, aesthetics and so on), we fail to see in what way the problem is also, perhaps even more so, one that concerns our motives and desires, and our capacity for acknowledgment as much as for avoidance. In other words, our obsession with knowledge makes us blind to the ways in which the problem is above all ethical. Thus, the crux of the matter, it seems to me, is why we, anthropologists, for all our care, trained attention and goodwill, are so often inclined to avoid grappling with the issue of how scepticism is *lived*.

I will draw inspiration mainly from Stanley Cavell's way of taking up Austin and Wittgenstein's answers to scepticism, ‘both with respect to whether we can know that a world of things exists at all and whether we can know that there are other minds, creatures who share our capacities of consciousness, who are aware of us as we are of them’ (2003: 21). Cavell's characterization of scepticism in terms of a certain mood – of worry, fascination, exclusiveness, obsession or fantasy, for instance – by which we are drawn to certain conclusions and incited to act in one way or another, is of particular interest here. Not all questions are alive for a person at just any time. I am not always in a position of asking ‘…why it is that something exists rather than nothing, or whether we can know that we are not now asleep and dreaming that we were awake…’ (Ibid.: 26). There are moments when these questions may be important; others when they do not arise or are simply empty. But when such questions trouble us, we are likely to indulge certain conclusions rather than others. For example, I might be tempted by solipsism if I am in a mood of ‘worry about whether I ever know another is in pain’ (Cavell, 1999: 79). Or I might be attracted by the idea that we each possess a private language (inaccessible to others) if my mood is to ask whether we can access another's interiority. Or else, we might give way to the thought that we cannot make sure that *any* object exists *at all* because we’re in the mood (of cultivated or forced astonishment, or suspicion) of doubting whether we can know that *that* object (i.e. that table) exists. In what follows I would like to look closer at how a certain mood of asking questions (an attitude, a frame of mind) encourages a drift towards scepticism that precisely takes the form of a claim against scepticism. This way, I hope to make clear that such a drift is prompted by a certain use of language. How do our anthropological texts sound?

## The inaugural scene

Let us first consider the opening scene of the book, which Bubandt defines as his ‘arrival story’, meant to introduce the reader to ‘the impossible experience that witchcraft constitutes in Buli’ (2014: xv), the village on the North Maluku Island of Halmahera where he inquired. It features him alone in the dark, one night of June 1992. ‘It was well past midnight’ (ix) and he could not find sleep. As he was fine-tuning his radio to distract himself, he ‘heard something outside the house, right outside the corner post next to [his] bed. It sounded like a dog…’ (ix). Unlike the usual sound of a dog gnawing on a juicy bone, ‘there was something out of the ordinary about the sound of that particular dog’ (ix). Even more oddly, the chewing appeared to come from the rooftop, as if the dog had climbed one of the shack's posts. His mind had difficulty getting around the matter. The tension rose. His perception conflicted with his reasoning: it was ‘impossible,’ for ‘dogs cannot climb vertical slopes’ (x); and even if one nonetheless had managed to do so, it would have fallen straight through the sago-leaf rooftop. So, he ‘listened for a while,’ until he decided to grab a ‘flashlight in one hand and a machete in the other and went outside.’ But he ‘found nothing suspicious,’ so he ‘went back inside’ and ‘into bed’ again. ‘There was definitely something strange, even eerie, about this dog that [he] could not find.’ (x)

At that moment, Bubandt was thinking of the figure of the *gua*, which he defines as ‘cannibal witches who attack people to eat their liver – but [he] had always shrugged them off’ (x). His interest in them, he admits, was first purely professional and intellectual (driven by his ethnographic endeavour) but was not relevant (or so he thought) to his existence or to the conditions of his field experience. However, ‘when [he] was caught in the dark…’ something about the *gua* became ‘suddenly all too real’ (x). The *gua* became tied to his existence and put him at stake, when he started to realize that the human beings with whom he was living – ‘fellow villagers, neighbours, family, or friends’ (x) – the ordinary people around him, could be forced by greed into an alliance with a *gua* and, at night, turn themselves into a fierce spirit, which could come in the shape of a cat, a wild pig, a bird or a dog: they would then ‘prey on other people against whom the host holds a grudge’ (x). Bubandt's growing awareness that ‘*gua* witches preferred to attack people who were alone,’ made him reconsider his friends’ pieces of advice to install electricity (for light) and not to live alone. But still, he had insisted on remaining alone in his private home,^3^ now in the dark, and was ‘beginning to regret [his] elective solitude’ (xi).

A few minutes after the chewing sound had disappeared, it came back ‘even closer, louder. In the darkness, panic set in. ‘Maybe the dog is inside the house!’ he remembers thinking (xii). Again, he snatched the flashlight and checked under the bed. There was nothing. He went back outside: there was still nothing to be seen. He writes, ‘Even as I mustered all my critical reason, I could not deny the tingling sensation of my spine. The invisible *gua* was bodily real in a way that all my doubts about its reality could not dispel. And yet I wanted to stand my ground, in and with this house…’ (xii). Indeed, the house had become his ‘comfort zone,’ his ‘bastion of privacy,’ where he could ‘withdraw to write notes…’ He called this place his ‘sanctuary,’ where he ‘could be an anthropologist’ and ‘maintain some semblance of personal privacy and professional identity’ (xii).

That night, the queer sound did not return. Still, he could not get to sleep, for its absence made it all the more present.

The next morning, Bubandt mentioned what happened to his adoptive family. Selena, the mother, was adamant: it was a *gua*, and preying on us is exactly what they do. Kenari, the husband, seemed more dispassionate about it: ‘Maybe it was a *gua,* and maybe it wasn't.’ Yet he still warned: ‘But you must have to be careful, Nils.’ The anthropologist's recklessness might put him at risk; but perhaps, also his hubris and tightness, as well as his too naïve generosity could harm him: ‘Don't act like a big boss, ordering people around while remaining tight with your money. On the other hand, don't give your heart [literally, ‘your liver’] to everyone indiscriminately.’ (xiii) Bubandt was then asked to take care, and act with tact.

The following week or so, Bubandt slept in Kenari and Selena's packed house. They all agreed that this was ‘better.’ (xiv)

Bubandt borrowed the house he lived in from Alena, one of his adoptive sisters. She moved out temporarily with her husband to her father's house during her pregnancy. The reason why she left, unknown to Bubandt at the time of the event, was that she had repeatedly encountered threatening spirits around the house and was afraid that a *gua* would eat or steal her foetus. Now, it was Bubandt's turn to be chased out of home by threatening spirits; and as a consequence, he went back to his adoptive family and was asked to learn more about local ways of behaving. Kenari's warning took the form of a scolding, and a reminder of what matters in this particular community. Nils, by his own admission, needed to show more tactfulness, for he had not yet the right measure of things: he had not found the ‘delicate balancing’ between the two tendencies of being either ‘*completely* taken in by all demands of reciprocity’ or ‘*entirely* extricating oneself from them’ (206, my emphasis; note the superlative use of adverbs, and his picture of the balance as that which is between two extremes). ‘Acting like a big boss’, ‘ordering people around’, ‘remaining tight with the money’, and ‘giving one's heart (liver) to everyone’ may well not be such adequate modes of behaving, according to locals. One is supposed to ‘make oneself small’ and ‘bow down’ as signs of respect (200); it is also ‘the best protection against witchcraft.’ (201) Thus, Bubandt's behaviour may put him in jeopardy if he goes on; he will not be protected, and the *gua* may be attracted (if not already).^4^

This scene is characterized by at least two movements. First, Bubandt ventures from the inside to the outside, and takes refuge back inside; but the threat then seems to follow him because it then comes from under the bed (note the analogy with a childlike fear). Second, he moves from aloneness (and isolation) to society (and reunion). These movements are two particular inflections of a movement of back and forth between privacy and public life, which are also part of the braiding of the familiar and the unfamiliar. They bear some relation to the fact that there is something uncanny next door, at home, or in the interstices of the home^5^ – i.e., at the threshold of the public and private – which not only unnerves but also puts pressure on one's thinking. ‘Maybe the dog is inside the house!’ His intellectual bewilderment that the outside is inside and vice versa seems to be generated by his epistemic posture that conceives the public and the private, the inside and the outside, as opposite and mutually exclusive terms. It is thus not so surprising that he is all the more fretting as he tries to maintain each hermetic to the other. A certain experience and image of the relation between the inner and the outer is at stake here, where the former is perceived as secure whereas the latter is perceived as threatening. Interestingly, the movement of the gnawing jaw is quite precisely delimiting the perimeter of the house. By the same token, it demarcates a structure of thinking that seems to hold him captive^6^.

Bubandt's response to the pressure is to grab in one hand a flashlight and in the other a weapon. On one side, he's anxiously trying to shine light in the darkness, to elucidate something in the world. On the other, he dreads what he may find and what it will do to him. Yet it happens as if it is precisely his drive to knowledge that produces his fear of it; or, in other words, his search for evidence produces the uncertainty over which he frets. Bubandt finally makes what seems like the right move: he goes back to his adoptive family and accepts being in the community. Only then do things get better. However, it is not over, for the lingering anxiety is somewhat still present, perhaps even more in the other's absence.

To what extent did Bubandt perceive the relation between his desire for privacy and his anxiety about the existence of the threat? His particular way of spinning the conceptual web of his thinking (that I will lay out in a moment), together with that which is not acknowledged in the book and which nonetheless is essential to the treatment of the problem of scepticism, generates a confused portrayal of scepticism, whereas he aims at precisely elucidating it. Paying heed to the form and the tone of his questions and remarks should, I hope, show us how almost unnoticed slippages in language can lead one, however unwittingly, to speak outside ordinary language games, doing so in a sort of void, where our questions and problems do not quite touch reality anymore.

## The mood in which the problem is posed

Bubandt's concerns are explicitly ‘epistemological and ontological’ (2014: xv). In particular, he highlights the ‘contradiction, patent to all in Buli,’ between what he takes to be an ‘ontological reality’ of the *gua*, on the one hand, and the fact of its absence, or ‘the impossibility’ of it, on the other. Witchcraft is also described as an ‘analytical topic’ (24) and, thus, is logically subject to analytical procedures (12) but baffles the analysts because it is thought to be both real and impossible (32). If there is undoubtedly a corporeal and experiential reality of witchcraft, he says, doubt is cast on it because the *gua* itself is ‘impossible.’^7^ In what way – logical, metaphysical, experiential, or else – we are to understand it as impossible, he does not say, but he insists on the contradiction that ensues: witchcraft is simultaneously irrefutable and fundamentally inaccessible. Here too, he does not say, to whom or to what it is inaccessible, but takes it as the main ‘unsolvable paradox’ (60). Thus, the problem is posed as one of argumentation and of accessibility: one cannot debunk the reality of the *gua*, but there is a ‘necessary doubt’ about whether one (the researcher and the natives alike, according to Bubandt) can access it at all. ‘What if witchcraft was problematic because its reality was simultaneously inescapable and unknowable?’ (20) Part of his endeavour is to unsettle the well-known theories – those of Evans-Pritchard and some of his followers – that have tried to domesticate witchcraft into explanations (7–12). He wants the reader to stay with the difficulty and to not give in too easily to the temptation of side-lining the problem, insisting that there is precisely no meaning or explanation to be sought. So what?

The idea he wants to debunk is that witchcraft is about beliefs. Instead, he suggests viewing it as an ‘experiential aporia,’ (xiv) a dead end of sorts. In a series of ‘theoretical excursus’ into deconstructionist territories, Bubandt draws inspiration mainly from Jacques Derrida, from whom he borrows, in a ‘toolkit approach,’ concepts^8^ such as ‘aporia,’ ‘hauntology,’ or ‘auto-immunization,’ which he then puts to the test empirically, ‘trying to probe the conditions of possibility and impossibility of experience’ (2016: 521). The main concept he is interested in is that of ‘aporia,’ which allows him to put into words ‘an experiential conundrum that has no resolution and that cannot be determined… Aporia marks an impassable situation, where understanding and the will to knowledge fail.’ (6) It is the point at which one is not able to go on anymore, where one is blocked, where there is no way, which makes the experience ‘painful and troubled.’ (6) Following Derrida's strand, Bubandt qualifies the *gua* as an ‘absent presence,’ whose ‘being’ (ontology) is to be accounted for through the study of the agency that arises out of the restlessness of doubt. His aim is to ‘explore the many forms of doubt that live beyond the Enlightenment’ (18). However, here, too, what the doubts precisely are about, and how we are to be answerable to the very different kinds of questions raised by very different kinds of doubts, he does not say. Having doubts about the existence of God or the validity of my argument; or being unsure whether it will rain; or doubting the loyalty of a friend, are all surely doubts of very different kinds and elicit very different kinds of responses.

His rather abstract point about doubt is coextensive of his picture of reality, which he qualifies as necessarily ‘undecidable’ (2) (another Derridean trope) without telling the reader how to understand such a claim. What are the terms of the undecidability? In what sense are the concepts of ‘reality’ and ‘decision’ related to the matters he is concerned with? If it may be the case for a theory, or a proposition, or an axiom, that it can be undecidable, i.e., that it cannot be proved or disproved, or confirmed or refuted, can it also be the case for reality? What would it mean to say so? In what sort of circumstances would it make sense to say that ‘reality’ is proved or refuted? Is this something the people in Buli are concerned with? We are left wondering. In any case, reality is said to be containing the intractable problem of witchcraft, of which supposedly we cannot make sense, and this puts human beings into perpetual unrest. ‘When it comes to witchcraft, nothing is certain… Humans can only guess’ (xiv). The ‘doubt’ is thus thought to be a ‘radical doubt’, which then prompts him to imagine ‘the ethnographic problem’ this way: ‘why is witchcraft experienced as proliferating in Buli if it has no social function, makes no sense, and explains nothing’? (14)

The radicalness of the doubt makes him consider that the anthropological problem is ‘the fundamental opacity that characterizes the Buli notion of subjectivity and sociality’ (30). ‘The minds of human beings’, he writes, ‘are fundamentally opaque to each other and to themselves’ (30). It is in this opacity that witchcraft is said to operate and out of which it is constantly reproduced. What interests him in particular is (1) ‘the uncertain reality of such absence and impossibility’ (xii); (2) that the evildoer, the ‘other may in fact be like oneself;’ and (3) that the ‘attempts to deny, combat, and eradicate… witchcraft in Buli appear to reproduce, historically and socially, the very problem they seek to banish’ (xii). Now, to clarify the sceptical problem as found in Bubandt, we need to articulate the ways in which the problem of knowing the other is related to the problem of knowing oneself.

## Echoes of scepticism in anthropology

Bubandt stresses from the very beginning that ‘the other may in fact be like oneself’ (2014: xii) and that ‘anyone could be a witch without knowing it’ (2). He did in fact pick up on the duality of the problem of scepticism about other minds. How does it appear?

First, Bubandt's problem – which he says is drawn from the Buli people themselves – appears as follows: since we postulate that the other's interior – one's feelings, sensations, intentions, thoughts, and so on – is inaccessible and out of sight, we must accept that this person's interior is impossible to know; and if we come to that conclusion, then we find ourselves in dismay, isolated from each other, alone facing dead ends. This strongly resembles the classical formulation of, and anxiety about, the problem of scepticism about other minds. However, what gives one the feeling that to ensure certainty, one would have to ‘get inside’ another mind, or at least see through the body of the other (which hides the mind)? What creates the sense that something is in the way? What is the urge that prompts one to believe that one needs to access something *beyond* something? That the problem is conceived as one of *accessibility* is a function of one's having drawn a certain picture of the relation between the mind and body, and of the inner and outer, where the body is seen as possessing the mind (which is trapped inside it); the consequence of such a view is that the body is seen as an obstacle, or a veil. The problem of knowledge, consequently, is posed in terms of finding ways of penetrating another's body (as if through a screen) to know what is hidden by it (i.e. the mind). The problem here is precisely that of the philosophical scepticism about other minds: the problem is getting *inside* another's mind to know it; thus, the body of *the other* is seen as an obstacle to my knowledge of the other.

Second, Bubandt turns a particular question – what can I know about another? – into a general, hyperbolic, doubt: How can I know *anything* about another?^9^ His language is often marked by a superlative form, as when he writes: ‘In truth, *nothing* about the *gua* is certain’ (2), ‘Witchcraft is *always* bad’ (4), or ‘Sociality is *always* magical’ (206, all my emphasis). In a later reply to Pelkmans’ (2016: 501) invitation to reconsider Wittgenstein's point about doubt and certainty – that certainty, uncertainty, and doubt are grammatically linked in specific ways, that one cannot exist without the other – Bubandt writes: ‘It seems to me that the uncertainty *about any given* instance of witchcraft (the empirical) points to, recalls, brings into implicit frustrated and anguished discourse the unknowability of *every* instance, and thus of the *general* (or *transcendental*)’ (2016: 523, my emphasis). Language itself seems to have been carried away.

I have written earlier that there is often a certain confusion between the two sorts of scepticism. It seems to me these passages are examples of such confusion and what it entails. On the side of scepticism about the outer world, the problem of knowledge is a *general* problem that concerns the world (or reality) as a whole; my knowledge of the existence of *all* things is at stake (if I can doubt that *this* table exists, then I can doubt that all tables – and by extension all things – exist). On the side of scepticism about other minds, the problem concerns *particular* other human beings; my doubts concern another individual with whom I am in relation, not a generic object. This means that my knowledge of *that* person cannot be extended to all others (the fact that, for instance, Othello can doubt Desdemona's trustworthiness does not imply that he doubts about the trustworthiness of all women or all human beings). However, Bubandt's conflation of the two inflections of the sceptical problem, subordinating the latter to the former, feeds through to his perception of the problem in Buli. What appears to him to be the core matter is: *because* the other's intentions (or inner thoughts) are at some point unclear to me, I *cannot* know the other *at all* (he or she is ‘essentially unknowable’), and it is *because* I doubt whether I could myself be someone I do not recognize (a *gua*) that I do not and *cannot* know *anything* about myself.

Now, can he really mean what he says? Chapter 7 begins with a quote by his friend Kenari: ‘It is difficult to know the inner core of people.’ Then Bubandt starts his paragraph this way: ‘Over the years, I had gotten to know Lolos [another friend] fairly well.’ (180) So, he *does* know others and some things about them (others like Lolos, who is accused of being a *gua*). How can he say at once that he knows Lolos and claim so insistently that the other is unknowable? What does this internal contradiction reveal? There is a significant, even crucial, difference between underlining the difficulty in knowing one another, as Kenari did, and stating a general, transcendental unknowability, as Bubandt does. It seems like he takes a step too far when he moves from the difficulty to an impossibility (the logic being: we cannot secure our knowledge of the other, *therefore* the other is unknowable). The problem with the ‘*therefore*-arguments’, justly pointed out by Cora Diamond (2003), is that they move from ordinary observations and expressions of difficulties ‘to conclusions that are presumed to compel rational agreement of all reasonable persons’ (MacArthur, 2020). In abstracting away from our embodied experiences, arguments tend to deflect us from the appreciation of the difficulty of reality. By converting the difficulty of coming to know another person into an unanswerable metaphysical problem or a theoretical impossibility, we run the risk of denying at once that person, what she expresses, and our own answerability to that other. This amounts to denying something about ourselves. Bubandt's obsession with figuring out what witchcraft *really* is leads him to take certain leaps that Kenari does not.

In another discussion, Bubandt asks, ‘What is the *gua*, really?’ Kenari replies, ‘This is what the police also ask’ (42). Bubandt misses the lesson in Kenari's response, and also that in Kenari's response we find a crucial difference that, if recognized, would undermine Bubandt's theoretical claims, and probably change his whole attitude towards the problem. For this is precisely where scepticism sneaks in: when we start to think that because *some* things are uncertain, then *nothing* is certain, we indulge the sort of slip that feeds our flight out of the ordinary; we have lost sight of the fact that when we say ‘there is nothing we cannot say,’ that does not mean ‘we can say *everything*,’ and when we say ‘there is nothing we cannot know,’ that does not mean ‘we can know everything’ (Cavell, 1999: 239). Yet, Bubandt's treatment of scepticism about the external world and scepticism about other minds as being roughly the same precipitates his question of whether we can know anything about others at all. But there are important differences to consider. There is no symmetry between the two forms of scepticism (Goodman, 1985).

There is a particular difficulty with the problem of scepticism when it comes to other minds, that is characteristically bypassed by those who treat it the same way they treat the problem of scepticism about the external world, which is the specific dual structure of the problem of other minds (Cavell, 1999: 442). Bubandt's scepticism is construed as one primarily concerned with what he (I) can know of another, not what others can know of him (me), although this duality does appear from time to time. Consequently, his way of conceiving the problem of otherness misses the fact that it ‘is exactly as much a question about me as about anyone else. If *anyone* is an other mind, *I* am one – i.e., I am an other to the others (and of course others are then I's to me)’ (Cavell, 1999: 442). If we acknowledge this, then the question becomes one of responsiveness.

Yet, the question Bubandt seems to express is one of intellectual limitedness, as if the problem was that we were always running short of tools, methods, models, and concepts to solve the puzzle. The sort of restlessness that characterizes such a mood makes one drift away from the shores of the concrete ordinary, for when one thinks that the obstacle is one's intellectual shortcomings, then one may feel as though one were being warded off from reality. Thereupon, the consequence is often that one feels the urgent need to access reality (as if one were outside of it), a reality of which other minds are part and parcel; these too are felt as having to be entered in order to be known. Bubandt's tendency to turn ‘the condition of humanity, into an intellectual difficulty, a riddle’ (Cavell, 1999: 493), that is, to make the problem intriguing, or beguiling, is more generally part of the deconstructionist kind of tone, where a certain mystery – thus attraction – hovers around the phrasing. It is precisely Bubandt's use of ‘aporia’ that prompts him to imagine his problem as a ‘puzzle’ that has the form of an impenetrable ‘enigma’ (2014: 35). The picture of witchcraft's main condition as one ‘of being-hidden’ (37) surely tempts one to look for it; it excites the imagination. Yet this picture is exactly what nourishes his scepticism, his need to go beyond the surface (or the screen, veil, skin, or appearance) and to imagine that transcendence will allow him to know what is beyond. Is he bewitched by witchcraft? Ironically, the very concept of aporia originally used by Derrida to deconstruct Western metaphysics ignites Bubandt's metaphysical escape out of the ordinary.

One could read such escape also as a refusal to be known (or as a desire for privacy). For Cavell (1996), in contrast to Derrida, knowing the other is related to the question of how I make myself known. The fantasy of a private language and inexpressiveness ‘would relieve me of the responsibility for making myself known to others – … it would suggest that my responsibility for self-knowledge takes care of itself – as though the fact that others do not know my (inner) life means that I cannot but know it’ (Cavell, 1999: 351). What Cavell means here is that self-knowledge cannot even be conceived, let alone formed, without others, without the others’ words that are also mine. My responsibility, then, does not ‘take care of itself’, but it is an everyday work of making myself known, intelligible, to others^10^ (also Das, 2020).

One can fathom the importance of the duality of the problem by looking at how knowing others is grammatically related to knowing oneself. An important dimension of our knowledge of others is their knowledge of us and that which we learn from their knowledge about us. More importantly, our relationships are staked in the ways in which we recognize or deny each other. It is not casual that these questions arise in the regions of human experience of pain. Questions about our relationships emerge with a particular acuity when one confronts the suffering of others and thus, our own. The question is no longer ‘how do I know that the other suffers?’; instead, it becomes, ‘how do I respond?’ A claim is made upon us, asking for acknowledgment and to which we are invited to respond. However, the claim may go unanswered (Cavell, 2015: 220–45).

## Slippages in language

Bubandt describes in detail the complex consequences of a series of alleged *gua* attacks and related deaths. One of his friends, Lolos, and his sister Yantima, were accused by villagers of being responsible for many of the *gua* attacks. They underwent enormous pressure: intimidation, death threats, assaults, and attempted murder. The deep and long histories of resentment between families (dating back at least to the 1930s) got out of hand. Lolos’ house was pelted with stones, and on several occasions, mobs broke into his house with the intent to kill him, in response to which Lolos sued some of his assailants. A petition was filed and signed by most of the heads of families, as well as the village head and the minister, to oust Lolos and his family. The tension was such that police, backed up by army officers, would have had to be almost permanently present to enforce safety. Eventually, the authorities convinced Lolos and Yantima to move.

One consequence of all this is that Lolos started to doubt himself, about whom or what he was. He and his family now had to deal with the following question: Was Lolos actually a *gua* after all, despite himself? And were the others too, without knowing, also *guas*? During several years thereafter, they took steps to solve this troubling question. At this point in the story, Bubandt relates in the style of free indirect speech Lolos’ thoughts: ‘If other people are essentially unknowable, am I also unknowable to myself? Can I be a *gua* and not know it?’ (2014: 182).

I have four observations to propose here. First, not being transparent to others and to ourselves is a basic condition of human existence; it is a feature of our ordinary life as human beings (Cavell, 1999; Das, 2020; Han, 2020; Moran, 2001; Motta, forthcoming; Wittgenstein, 1986). But this does not mean that we’re always totally opaque; there are things we do know about others and ourselves. Hence, to move from invoking the fact that it is unclear to me who I am (are we ever clear on that?) to stating that I am essentially unknowable, is a metaphysical shift we might want to avoid. Second, our capacity to claim a voice, hence, to be someone, a person, say, Lolos, is dependent on the capacity of recognition of that voice by others. If we look at how Lolos responds to the suspicions that others raised about him, it seems to me that part of his problem (or anxiety) is: How can he make himself known to those others who do not acknowledge him? If he surely had to fight ‘a frustrated battle to reconcile, in his own mind, *what he knew about himself* with what *everyone else* was saying about him’ (2014: 180, my emphasis), he was also concerned with giving public evidence of his innocence, as if, as a last resort, what is left is to try desperately to prove one's existence. So – and this is my third point – the problem is not that he ignores things about himself, but that the others do not receive and accept what, and who, he is. ‘Lolos, *as far as he himself knew*, was not a *gua.*’ There is a ‘*hesitation* about his own knowledge of himself’ (185, my emphasis). So, he is *not* unknowable after all, as Bubandt claims. Furthermore, it is untrue that ‘everyone else’ is supposing he's a witch, since Kiama, his wife, and Yantima, his sister, support him, as well as a ‘few other people in the village.’ This state of fact cannot simply be ruled out. It matters that they trust and help him, not only to search for a cure, but also for proof that he is not a *gua*. Thus, there are people who actually do know that Lolos is not what others accuse him of, and who care. Not everyone becomes a *gua*, certainly not under all circumstances. It most often has something to do with the ‘dark side of kinship’. Bubandt himself explains that there is indeed a certain milieu, a context, and contingencies for becoming a *gua*. In Kenari's words: ‘The pit falls from parents to children, and some of them will get it. That is reality. Would you not say this is difficult, Nils?’ (51)

My final observation is that it is precisely Bubandt's nonrecognition of the points above that allows him, regardless, to infer that ‘the uncanny potential of witchcraft’ lies in ‘the possibility that *anyone* can be a witch’ (182, my emphasis). Grammatically, this includes Bubandt himself; but he seems not to consider himself part of the picture. So, is it ‘anyone’ but him? Or is it that this possibility does *not* concern just *any*one? Regardless, such ‘sceptical self-appraisal’ (182) is imagined to be the effect of the ‘opacity of mind’ that apparently characterizes Buli epistemology. What is troubling to him is that ‘one can never know’ what sort of sentiment (anger? envy? something else?) the other harbours in relation to ‘me’. What is even more disconcerting for Bubandt, is that one may be ‘unable to know the inside of one's own mind’ (182), something he conceives of as the ‘uncanny corollary’ (183) of the opacity of other minds.

The language used signals a slight excitement about what appears to be a discovery, or a fascination with an ‘enigma’: ‘witchcraft, *essentially*, is inaccessible to the human mind because the human mind is inaccessible to itself as well as to others’; ordinary sociality is ‘beset by the *radical* alterity that is witchcraft’ (183, my emphasis). One notably feels the excitement by the repetition of the words ‘opacity’ and ‘uncanny,’ put in relation with the vocabulary of cannibalism, all of which hints at a world of darkness and creepiness. These are important features of the Gothic narrative. In other passages of the book, ‘opacity’ is put in relation with the concept of ‘horror’ (of the *gua*, thus of oneself), hence giving darkness another tint, one of gloominess, fierceness, tenebrousness, and perversity.

I am not denying that these are the tints perceived by Bubandt. What I am saying, is that one can easily be swept away by the fantasy they arouse, and not see any more that what is before us is, as Poe writes in the outset of *The Black Cat*, a ‘series of mere household events’ (1984: 1135). It seems like it did not occur to Bubandt that Lolos might have been suffering from the very fact that he was not recognized for who he was (Lolos), and because he was not acknowledged for what he was (a fellow human being, not a *gua*). The suspicion eventually chiselled a wound, to the point that it breached his own perception and knowledge of himself, sowing in him a ‘lingering, nagging doubt’ (184). We should not forget that he was harmed by the (violence of the) denial of the community. Lolos eventually died from a disease in 1999. After Lolos’ death, new suspected witches surfaced, ‘who came from families that, only a few years earlier, had been among those accusing Lolos of witchcraft, an irony that Lolos’ family was quick to point out’ (230). In this context, it seems like the *gua* expresses the threat of scepticism. And this is now part and parcel of the world's knowledge: Lolos, Yantima, and Kiama know something about what it is to be singled out and outcast: thus, they know what life is like under the threat of being killed. ‘Scepticism is lived’ (Cavell, 1999: 440); lived as a lethal potential.

A distinctive feature of Bubandt's way of proceeding is that he rushes ahead when precisely there is a need for pause. For instance, this is the case when he lays bare the contradiction between the ‘impossibility’ of witches and their dangerousness: ‘The conditions of knowing anything about witches, is always fraught at best and riddled with contradiction’ (xiv). He then spares no effort to try to make sense of it by forcing irreconcilable terms into conciliation. The experience of witchcraft is said to be ‘exceedingly corporeal’, even visceral (the *gua* eats your liver or foetus), but at the same time, it is depicted as inaccessible to human senses and understanding (xiv). Now, the fact that reality can, at times, and under certain conditions, appear to us as unreal or surreal, or contradictory, is an ordinary fact of our living as humans, and no doubt a dimension of what reality is (Benoist, 2021; Lambek, 2021). I do, of course, not call into question that Bubandt feels all the more troubled by this very unreality as it ‘exacerbates its menace’ (2014: xiv). I have no doubt he has gone through difficult hardships and, as he says, that the fear is contagious. I do not question either that the figure of the *gua* inspires disgust, terror and panic among some people he met. Moreover, this feeling that our sense of reality becomes particularly alive precisely when reality seems to be vanishing is not unfamiliar to me; I elsewhere (Motta, 2019) suggested it could be taken as a point of departure for an investigation of our concept of reality. In another paper (Motta, 2021) dealing with the thrill generated by the clichés around the figure of the zombie, I have asked how important it is to pause and consider the question of what it would amount to, to give a realistic account of such matters. This question is critical for the case at hand because the feelings of dread and revulsion that Bubandt describes when he's concerned with the *gua*'s perversion – our horror of it, that is, our horror of us – push him to qualify the *gua* as abhorrent, disgusting, abject, horrible, and intolerable (Ch. 5). What does it revert to – and what does it say about us – that we say these things about others that are suffering human beings, our neighbour, a friend, a sister or ourselves?

Rosalind Morris (2016: 515) justly draws our attention to the fact that ‘not all doubts engender fear, and not all fear is born of doubt.’ It seems that there is a risk of being carried away by the movement of one's argument, and, thus, that we lose touch with the everyday reality of those with whom we share our lives. The sense that there is *always* a lingering violence waiting to erupt behind witchcraft doubts seems to be derived from such a loss. Utterances about witchcraft are surely not always expressions of doubts and hence, do not always give way to violence, but they can also be a language for our sufferance (Luongo, 2010), or just ordinary ways of relating to each other, such as jokes or rebukes or insults. Bubandt himself gives an example. One morning, Kenari's daughter Lina was bathing her baby who was unhappy and squirming. Impatience grew in Lina until she ‘raised her hand in mock threat, as if ready to strike the child’ (2014: 192). Kenari, who was drinking coffee with Nils, ‘turned to his daughter and admonished her: ‘You’ll become a *gua*, Lina!’’

In the book, the shifts in language (in mood) that are prompted by a certain fascination with matters we do not quite wrap our minds around or that resist our desire to grasp are partly due to what Wittgenstein (1986: §109) calls ‘the bewitchment of our intelligence by means of language.’ Something in our enthrallment has made us quit the rough ground of ordinary language, as if we were at some point not capable anymore of speaking ordinarily, or unable to recognize our ordinary words (like, for instance, when I say: ‘I know her, she means it!’ or, ‘You’re right, I resent him’). Despite an undeniable opacity of our experience of the world, of others and ourselves, we actually *do* know things about others’ intentions and thoughts and feelings and about ourselves; and often, others teach us something about ourselves we did not know.

Wittgenstein is known to have conceived of his philosophy as a cure that takes the form of a return to the ordinary. In response to our metaphysical flights, we need to learn to speak our own language again because there always comes a point where we lose touch with our words; and such a loss can be crippling and damaging (Cavell, 2010). Wittgenstein's deep insight is that our initiation into our forms of life is never achieved once and for all; it is never-ending. The battle against our bewitchment is one against our own inclination to be misled by means of language. Hence, what other choice do we have than to reclaim our words when they have gone into exile and left us stranded? As it happens, at times, we lose them (Diamond, 1988); but then, our quest is to figure out a way to lead them back home or accept the loss. However, Wittgenstein cautions us: there is no ready-made and no definitive solution to this, no recipe that will guide us throughout our difficulties with reality. This is where an investigation starts.

## Anthropology's hopes

Bubandt's *The Empty Seashell* achieves something: it offers a stimulating entry point into thinking a particular form of human relationship – witchcraft – in terms of scepticism. It justly draws attention to the fact that others are not easily legible, and that we are not as transparent as it is sometimes assumed. This is rare enough to be praised. But it does more: it also shows that scepticism has a history and that the history of Buli generates a particular kind of scepticism which needs to be thought in its own terms. I am delighted by the incursions Bubandt makes into foreign regions of thought. His complex ethnographic material allows him to explore unconventional grounds and enables a refined discussion between anthropology and philosophy.

Yet, as I have shown, Bubandt's descriptions of the sceptical problem are not entirely satisfying. First, because he does not recognize that the problems he raises are raised in language, and thus that scepticism is lived, lived in language. The contradictions and paradox that he mentions are not experiential, as he thinks, but logical; they are an effect of the language used to describe the problem, not of reality. Reality is just what it is, nor contradictory, nor paradoxical, nor bad, nor good (Benoist, 2021). Along the same line, aporia does not apply to reality, but to our thinking; it is the theory (our ways of speaking), not reality, that generates aporia. What is fallacious or incongruous, is our way of perceiving and thinking the difficulty, the way we stand, ‘the frame through which we look’ (Wittgenstein, 1986: § 114). The same goes with his claim that others are unknowable in themselves; it is our posture or attitude towards them that creates the unknowability. Our desire for certainty will not be assuaged by looking for more knowledge. In other words, it is our wish to secure knowledge about reality (not reality itself) that generates the anxiety about the fact that it eludes us.

Second, my dissatisfaction concerns his preoccupation with epistemology and ontology. Despite all the clues Bubandt recognizes, he misses that at some point, witchcraft is also a specific language – an expression – that allows humans to speak out their concerns and express suffering. And when people in Buli shy away, it is itself relational, ethical. Bubandt turns that ethics into an aporia, and he reads it as their issue, whereas it results rather from his posture towards the problem. It seems to me that at times he tends to overreach, or misrecognize, because he heads towards analysis after all (also Bubandt, 2017). It is precisely by applying analytic procedures that one is inclined to think of these issues as issues concerned with competing truth claims (and not, say, with our capacity for acknowledgment); and that is precisely what produces the aporia (and thus blinds us to the ways in which the problem at hand is not aporetic).

I emphasized earlier the importance of the mood in which the problem is posed. Let me end this essay by highlighting the moods in which our anthropological hopes may be expressed. At the very beginning of Bubandt's book, the acknowledgments end with this phrase: ‘It is my hope … that one day the *gua* will be gone from Buli … without the shadow of a doubt.’ (xix) I suppose he means above all that he wishes for *accusations* to cease. Like his friends in Buli, he yearns for less violence and suffering. His wish is in some ways very natural; he expresses love for the people he cares for. But still, the ambiguity remains. Bubandt showed himself, the *gua* is not just something one wants to see go away; the *gua* is a man or a woman of one's community; it can be a neighbour, a friend, an uncle or oneself. It can be an accusation, an excuse, a rebuke or an insult. It can also be the perception of an ongoing threat, the sense of mutual distrust, the heralding of a catastrophe still to come. What then does such a hope say about where we stand as anthropologists and what we aspire for?

Imagine the inaugural scene recalled above as a scene of instruction that shows our human readiness to fight, our preparedness for battle, in moments when our desire for assurance is bewildered. It is as if we, humans, as restless and embattled creatures, are ready to wage war against one another. In parallel, Bubandt announces that the book historically traces ‘the attempts by Buli people to rid themselves of the *gua*’ (6), and he shows in detail how the modernization of the state apparatus and Christianization of the population ‘seemed to offer the hope of eradicating witchcraft’ (6), as much as it raised profound disillusions. Such a hope, of course, has a history that is inseparable from the history of the hopes placed in the Enlightenment project: it is as much a history of our disappointments as it is a history of the violence that came with Modernity.

Now, I cannot help but think of the colonial hope for, and illusions about, the disappearance of witches. And witches reappear and multiply all the more so as we consolidate our efforts to deny their existence. One thing seems sure enough: the wish for their nonexistence bears the seed of violence not always fathomed.

The disturbing affinity between the expression of Bubandt's hope and the hopes of Modernity must tell us something about the way we picture anthropology and what it is about. The quest, say the unquestioned drive, or the unquenched thirst, for knowledge may run against us. If witchcraft threats, as he recognizes himself in chapter 7, emerge from the opacity that characterizes our minds and thus our relationships – the shadows of kinship and friendship and neighbourliness – and that such darkness is not to be overcome, enlightened or resolved, as the electric lamp posts of Modernity promised (Ch. 8), then what kind of hope is the one he expresses, the hope to get rid of witchcraft? Doubtless, these are unsatisfiable cravings, but precisely because of this, they may also be dangerous ones.

## Notes

1. See Broughton, 2002; Dreyfus and Garfield, 2011; Garfield, 1995; Kjellberg and Ivanhoe, 1995; Kukkonen, 2010.

2. See, for instance: Brandel, 2022; Crapanzano, 2014; Das, 2007, 2020; Das, Jackson, Kleinman, and Singh, 2014; Duranti, 2008, 2015; Evans-Pritchard, 1976; Gable, 2002; Geschiere, 2013; Guyer, 2013; Han, 2020; Keane, 2008; Khan, 2012; Lambek, 2010; Pelkmans, 2013; Robbins and Rumsey, 2008; Robbins, 2008; Schieffelin, 2008; Schmidt 2017; Scott and Hirchkind, 2006; Segal, 2016; Siegel, 2006; Stasch, 2008; Taussig, 2003.

3. It is interesting to note that children turn up every evening to sleep with him, whether Bubandt likes it or not. What is their sense of the situation? What else could he have learned from them? After a while, they stopped coming; what does that tell us about what is going on?

4. Arrogance, stinginess, overindulgence and wrath are all mentioned in the book as the attitudes that make people more prone to becoming a *gua*. I recall the myth of Prometheus here. After having fooled the gods and stolen the fire to give it to human beings––which can be understood as having dared to hoax the gods and steal their knowledge––Prometheus is punished by Zeus who sends an eagle to eat his liver for eternity. The liver, conceived by the Greeks as the source of passions and emotions, especially anger and wrath, had the special status of being the seat from which divinities would speak: thus, it would be the organic locus of a special kind of divination, hepatoscopy (Derek, 2008). That the liver so often appears in so many different cultures as the source of anger should require greater attention, for it might tell us something about the naturalness of our human forms of life.

5. I am reminded of Poe's black cat walled in the house; or Madeline who, in *The Fall of the House of Usher*, is entombed alive in the family grave inside the house.

6. The structure of this scene is astonishingly resonant with a passage in the *Philosophical Investigations*. In §115, Wittgenstein (1986) writes: “A *picture* held us captive. And we could not get outside it, for it lay in our language and language seemed to repeat it to us inexorably.” The ending line of the previous remark (§114) goes: “One thinks that one is tracing the outline of the thing's nature over and over again, and one is merely tracing round the frame through which we look at it.”

7. It seems like the picture that holds us captive here is one that opposes the essence of reality (the *gua* itself) and our experience of it. This is roughly the classical realist or positivist kind of conception.

8. The authors of a recent collection of essays on concepts and reality have put into question the conventional assumption ‘that concepts are tools that enable us to translate between experiences, and in so doing, tell us something otherwise hidden about reality. The idea that concepts are tools, or equipment, for theory relies on an alluring conceit that they are the kinds of things that we are free to make and unmake more or less as we will, that we intentionally invent them (whole cloth, or in part) in order to discover or uncover something about our world, and without which we would be at a loss’ (Brandel and Motta, 2021: 3).

9. This generalized form of doubt is said by Robbins (2008) to be characteristic of the people in Melanesia and other places of Southeastern Asia.

10. This is also a point that Vincent Crapanzano, for example, brings under scrutiny in his meditation on scepticism in anthropology. During his fieldwork in Meknes in the 70s among the Hamadsha, he had to overcome, he writes, ‘the fear of being fully known,’ whereas he figured that he ‘can never fully know what they are experiencing’ (2014: 258). However, his way of addressing the issue is very different from mine.
